# Supplementary material for: Trustworthy diagnosis of Electrocardiography signals based on out-of-distribution detection
Source: PLoS One. 2025 Feb 25;20(2):e0317900. doi: 10.1371/journal.pone.0317900 (PMC11856293; doi:10.1371/journal.pone.0317900)
Supplement: Attachment — Submitted filename: Respondse to Reviewers the latest.docx [file pone.0317900.s003.docx]

Dear Editor and Reviewers,

Thank you for your editorial work on our manuscript PONE-D-24-31262, entitled " Trustworthy diagnosis of Electrocardiography Signals based on Out-of-Distribution Detection". We have double-checked our draft and have corrected any inappropriate content with the help of comments of all reviewers. The detailed responses to the reviewers’ comments in a point-to-point format are attached below.

These revisions do not affect our interpretation of the results, and we hope that the responses could be good enough for your editing.

With my best thanks,

Yours sincerely,

Zhibin Zhao (corresponding author),

School of Mechanical Engineering,

Xi’an Jiaotong University,

Xi’an, 710049, PR China,

E-mail: [zhaozhibin@xjtu.edu.cn](mailto:zhaozhibin@xjtu.edu.cn),

Tel./fax: +86 - 18717343671

**Point-to-point response to the reviewer’s comments**

We sincerely thank the reviewers for the careful and thorough review, which are quite helpful to make the paper more solid and fluent. We have revised our manuscript very carefully in the light of the suggestions and comments. The following responses are prepared to address all of the reviewers’ comments in a point-to-point fashion.

**(Comments in black, responses in blue, revised contents of manuscript in red)**

**Reviewer#4:**

1 - In the latest version of the manuscript, It is unclear how the threshold for ReAct is chosen. In an earlier version it appears the threshold is selected by a simple heuristic involving known ID and OOD samples. Is this still the case?

**Response:** We appreciate reviewer’s comments, which are very valuable and helpful for improving the quality of our paper, and the reply is as follows.

The threshold selection method in the current version remains consistent with the earlier version. Specifically, the threshold is used to truncate the activations in the penultimate layer of the network, limiting the upper bound of activations to reduce the influence of OOD samples on the model’s output. The selection process is based on the statistical distribution of ID activations. Details are as follows:

- ReAct determines the threshold based on the activation distribution of ID samples in the penultimate layer.
- The statistical characteristics of ID activations are calculated using only ID data.
- In the original ReAct method (as proposed in the paper "ReAct: Out-of-distribution Detection with Rectified Activations"), the threshold is typically set to a fixed percentile (e.g., the 90th percentile of ID activations), meaning that 90% of ID activations lie below or equal to this threshold.
- In our approach, rather than using a fixed percentile as suggested in the original ReAct paper, we conduct a grid search to determine the optimal threshold for the specific ECG datasets. Specifically, candidate thresholds are represented as different percentiles of ID activations (e.g., 5%, 10%, 15%, ..., 100%). For each threshold, we evaluate the model’s performance on the test set and select the threshold that yields the best experimental results.
- Figure 3 in the manuscript illustrates this process, showing an example where the activations are truncated at the 5th percentile.
- This truncation effectively removes abnormally high activations from OOD samples, thereby reducing their impact on the model’s output.
- For each of the three tasks in our experiments, we perform a separate grid search to determine the optimal threshold, as the ID activation distributions vary for different tasks.

Changes in the manuscript:

We have added the following explanation to the ReAct Mechanism section:

“The ReAct threshold was determined using a grid search approach. Specifically, candidate thresholds were selected based on different percentiles of the activation values from the ID dataset in the penultimate layer, ranging from the 5th percentile to the 100th percentile in increments of 5%. For each threshold, we evaluated the model's performance on a test set containing both ID and OOD samples. The threshold that minimized Detection Error (DE) while maximizing AUROC and AUPR was selected as the optimal value.”

2 - If indeed this is the case, then how well does the proposed method work when the threshold is determined using one set of ID and OOD samples, and is faced with a different set of OOD samples not seen during the threshold selection process?

**Response:** We appreciate reviewer’s comments, which are very valuable and helpful for improving the quality of our paper, and the reply is as follows.

The design of ReAct focuses on determining the threshold using only ID samples:

- The threshold selection process relies primarily on the statistical distribution of ID activations and does not directly depend on OOD samples.
- The fundamental assumption behind ReAct is that ID activations exhibit a relatively stable distribution, while OOD activations tend to exhibit higher variance and positive skewness. Based on this assumption, a threshold determined from ID data can generalize effectively to unseen OOD samples.
- In the original ReAct paper ("ReAct: Out-of-distribution Detection with Rectified Activations"), the threshold (e.g., the 90th percentile of ID activations) was shown to generalize well across a variety of OOD datasets (e.g., iNaturalist, SUN, Textures), demonstrating its robustness.
- In our paper, the grid search for the threshold is performed separately for each task because the ID samples differ across tasks, leading to variations in ID activation distributions. However, for a fixed set of ID samples, the threshold determined for one OOD scenario can also be applied to other unseen OOD scenarios without significant loss in performance.
- While additional optimization incorporating specific OOD samples may yield better results, the threshold selected using ID samples alone remains effective across various OOD scenarios.

In our work, we focus on combining ReAct with the energy-based method to enhance OOD detection in ECG data. The generalization capability of ReAct to different OOD datasets has been extensively demonstrated in the original ReAct paper, so we did not repeat these experiments but instead focused on its application to ECG tasks.。

Theoretical explanation on performance with unseen OOD samples:

1) Universal activation characteristics of OOD samples: ReAct leverages the common characteristics of OOD activations, such as higher variance and positive skewness, to improve ID-OOD separation. If unseen OOD samples exhibit similar characteristics, the threshold determined from ID distributions will likely remain effective.
2) Robustness of threshold selection: Since the threshold is based on the stable statistical characteristics of ID activations, its performance generalizes well to unseen OOD samples.
3) Limitations with atypical OOD samples: If the unseen OOD samples exhibit activation distributions that closely resemble those of ID samples, the effectiveness of ReAct may decrease. However, such cases are rare, as most OOD samples exhibit distinct activation characteristics compared to ID samples.

3 - Are the results obtained averaged over multiple runs (with different random network initializations, for instance) ? It is unclear how the results are obtained and aggregated.

**Response:** We appreciate reviewer’s comments, which are very valuable and helpful for improving the quality of our paper, and the reply is as follows.

Our experimental results are not averaged over multiple runs with different random initializations. However, we designed multiple experiments across different datasets and task settings to comprehensively evaluate the generalization and robustness of the proposed method. These experimental setups ensure that the reported results are representative and credible.

1. Experimental design:

We conducted experiments on two widely-used ECG datasets (MIT-BIH and INCART) with the following task configurations:

- **MIT-BIH Dataset**：
- ***Task 1:* [N, V, L] as ID classes; [A, R] as OOD classes.**
- ***Task 2:* [N, A, R] as ID classes; [L, V] as OOD classes.**
- **INCART Dataset:**：
- ***Task 3:* [N, V, R] as ID classes; [A] as OOD class.**

These tasks involve different ID-OOD combinations and cross-dataset validations to assess the method's generalization across diverse data distributions.

2. Reliability of results:

Although we did not perform multiple runs for each task, we ensured the reliability of our results through the following measures:

- Patient-independent data splitting:
- Data splits were based on patient IDs to prevent data leakage. For example, in MIT-BIH, 28 patients were used for training, 10 for validation, and 10 for testing.
- Cross-dataset validation:
- Independent experiments on the INCART dataset validated the robustness of the method across different data distributions
- Task diversity:
- Different ID-OOD configurations ensured consistent performance across varying scenarios.
- Data augmentation and regularization:
- Techniques such as Gaussian noise, random scaling, and dropout were applied to enhance robustness.

3. Future work:

We acknowledge that single-run results can be influenced by random initialization. In future work, we plan to conduct multiple runs with random initializations and report the mean and standard deviation to provide a more comprehensive evaluation.
