# Supplementary material for: Trustworthy diagnosis of Electrocardiography signals based on out-of-distribution detection
Source: PLoS One. 2025 Feb 25;20(2):e0317900. doi: 10.1371/journal.pone.0317900 (PMC11856293; doi:10.1371/journal.pone.0317900)
Supplement: Attachment — Submitted filename: Respondse to Reviewers.docx [file pone.0317900.s002.docx]

Dear Editor and Reviewers,

Thank you for your editorial work on our manuscript PONE-D-24-31262, entitled " Trustworthy diagnosis of Electrocardiography Signals based on Out-of-Distribution Detection". We have double-checked our original draft and have corrected any inappropriate content with the help of comments of all reviewers. Moreover, we have double checked the format of this manuscript and conducted more experiments to validate our proposed method. The detailed responses to the reviewers’ comments in a point-to-point format are attached below.

These revisions do not affect our interpretation of the results, and we hope that the responses could be good enough for your editing.

With my best thanks,

Yours sincerely,

Zhibin Zhao (corresponding author),

School of Mechanical Engineering,

Xi’an Jiaotong University,

Xi’an, 710049, PR China,

E-mail: [zhaozhibin@xjtu.edu.cn](mailto:zhaozhibin@xjtu.edu.cn),

Tel./fax: +86 - 18717343671

**Point-to-point response to the reviewer’s comments**

We sincerely thank the reviewers for the careful and thorough review, which are quite helpful to make the paper more solid and fluent. We have revised our manuscript very carefully in the light of the suggestions and comments. The following responses are prepared to address all of the reviewers’ comments in a point-to-point fashion.

**(Comments in black, responses in blue, revised contents of manuscript in red)**

**Reviewer#1:** This article generally has a well-structured writing style and adequately explains the research findings. However, the overall novelty of the article is low, as it mainly presents the integration of existing techniques. The experiments conducted are also insufficient to support the claims of novelty in this research.

**Response: We appreciate reviewer’s comments, which are very valuable and helpful for improving the quality of our paper. We will respond to your precious comments in a point-to-point fashion.**

**Comment 1:** Novelty

Assessment: Poor While the article presents an interesting combination of techniques for trustworthy ECG diagnosis, its novelty is limited as it primarily relies on integrating existing techniques. The main contribution lies in the application of this combination to the ECG domain, but it does not offer significant methodological advancements or deep new insights. Here are some detailed reasons why the novelty of this research is considered low:

• Use of Existing Techniques: The article essentially combines several previously established techniques:

o CNN and Attention Mechanism: The use of CNN for feature extraction from ECG signals and attention mechanisms to enhance representation learning is a well-established approach in the literature.

o Energy Score and ReAct: Although the use of the Energy score for OOD detection and ReAct for handling overconfidence is relatively new, both techniques have already been explored in other contexts.

**Response:** We appreciate reviewer’s comments, which are very valuable and helpful for improving the quality of our paper, and the reply is as follows. We acknowledge that the techniques we use, such as CNN and Attention Mechanisms, have been previously applied in other fields. However, the novelty of our approach lies in the specific integration and application of these techniques to the ECG domain for Out-of-Distribution (OOD) detection. While CNN and Attention are well-established, their combination with Energy Score and ReAct for trustworthy ECG diagnosis is a novel contribution. This unique integration allows our model to not only classify known heart conditions but also reliably detect unknown heart diseases, which is critical in clinical applications. We have clarified this point in the revised Introduction and Methods sections to highlight the novelty of our approach.

Changes Made:

1. Expanded the Introduction to emphasize the novelty of integrating CNN, Attention, Energy Score, and ReAct specifically for ECG OOD detection.
2. Updated the Methods section to detail the unique aspects of our technique in the context of ECG diagnosis.
3. As an example, we have added the following text to the Introduction: “While CNNs and Attention Mechanisms have been widely applied in other areas, their integration with Energy-based OOD detection and ReAct specifically for ECG diagnosis is novel. This combination allows the model to not only classify known heart conditions but also reliably detect unknown heart diseases, addressing the critical need for trustworthy diagnostics in clinical settings.”

• Direct Application to ECG: While the combination of these techniques for ECG diagnosis may not have been done before, the article does not provide strong evidence that this application requires significant methodological innovation. It is more of a direct application of existing techniques to the ECG domain.

**Response:** We appreciate reviewer’s comments, which are very valuable and helpful for improving the quality of our paper. In response, we have expanded the problem background to clarify why direct application of existing techniques to ECG signals is not trivial. ECG signals present unique challenges such as domain shift, noise, and class imbalance, which are not typically encountered in other applications. Our method is specifically designed to address these issues through the combination of CNN-Attention for feature extraction and Energy-based OOD detection to handle the presence of unknown heart diseases. This is particularly important in clinical diagnostics, where unknown heart conditions can have serious consequences.

Changes in the manuscript:

We have added the following explanation to the Problem Background section: “ECG signals are highly variable across patients due to differences in age, gender, and pre-existing conditions. Additionally, small variations in signal noise or recording conditions can significantly degrade model performance. Our proposed method addresses these challenges by combining CNN-Attention mechanisms for robust feature extraction and Energy-based OOD detection to identify previously unseen heart conditions.”

• Lack of Comparison with Other OOD Methods: The article mainly focuses on comparisons with traditional Softmax-based methods. A more comprehensive comparison with other state-of-the-art OOD detection methods in the ECG domain would strengthen the novelty claims.

**Response:** We greatly appreciate the reviewer’s suggestion to include additional comparisons. We have now conducted experiments comparing our method with ODIN[1], a well-known state-of-the-art OOD detection technique. These results are presented in Table 6, and we have provided a detailed analysis of the comparison in the Experimental Results section. Our method demonstrates superior performance in terms of AUROC, AUPR, Detection Error (DE), FPR95 and FDR, further supporting the novelty and effectiveness of our approach.

Changes in the manuscript:

The following text was added to the Experimental Results section: “As shown in Table 6, our method significantly outperforms ODIN in terms of AUROC and AUPR across all tasks. For instance, in Task 1, our method achieves an AUROC of 97.27%, compared to ODIN’s 71.18%, demonstrating the effectiveness of integrating Energy-based OOD detection with ReAct.”

1.	Liang S, Li Y, Srikant R. Enhancing The Reliability of Out-of-distribution Image Detection in Neural Networks. arXiv; 2020. doi:10.48550/arXiv.1706.02690

• Limited Focus on Anomaly Detection: Although OOD detection is important, the article primarily emphasizes identifying anomalies (unknown heart diseases). A more significant contribution could be made if the method also addressed the enhancement of known heart disease classification or offered new insights into OOD results interpretation.

**Response:** We appreciate this valuable comment. In response, we have revised the manuscript to place a stronger emphasis on how our method also improves the classification accuracy of known heart diseases. Specifically, the CNN-Attention mechanism enhances feature extraction for both ID and OOD samples by focusing on the most relevant parts of the ECG signal. We have also added more discussion of the Energy Score and ReAct techniques, explaining how they contribute to a more reliable interpretation of OOD results and reduce overconfident predictions on unknown samples.

Changes in the manuscript:

We have added the following paragraph to the Results and Discussion section: “In addition to improving OOD detection, our method enhances the classification performance for known heart diseases. The CNN-Attention mechanism focuses on the most salient features of the ECG signal, improving the model’s ability to differentiate between similar arrhythmias. Furthermore, the Energy Score provides a more reliable confidence measure, reducing the likelihood of overconfident predictions on both known and unknown cases.”

**Comment 2:** Problem Backgrounds

Assessment: Inadequate The article provides a generally clear background on the importance of ECG diagnosis and the challenges faced, particularly in detecting unknown heart diseases. However, there are some shortcomings in the problem background in the introduction:

• Lack of Depth in Existing Challenges: The article mentions that current deep learning methods struggle with OOD heart diseases but does not deeply explain why this occurs. A better understanding of the underlying technical challenges, such as domain shifts or lack of representative training data, would enrich the problem background.

**Response:** We appreciate reviewer’s comments, which are very valuable for improving the quality of our paper. We agree that the original manuscript did not provide a detailed explanation of the technical challenges faced by deep learning models in detecting OOD heart diseases. In the revised version, we have expanded the Problem Background section to discuss why deep learning models, particularly those using traditional Softmax classification, tend to be overconfident in their predictions for OOD samples. We have also included a discussion on the domain shift problem and the class imbalance issue, which are particularly relevant in ECG datasets.

Changes in the manuscript:

We have added the following paragraph to the Problem Background section:“This is because deep learning models, particularly CNNs and RNNs, are designed to excel at learning patterns present in the training data, meaning they can struggle when encountering data that deviates from this distribution.”“The difficulty in handling OOD samples stems from several technical challenges. First, domain shifts between the training and testing data—such as variations in patient demographics or different ECG recording environments—can lead to performance degradation.”“Class imbalance is another challenge in ECG datasets. Rare arrhythmias are often underrepresented, making it difficult for models to learn robust features for these conditions.”

• Limited Focus on Anomaly Detection: While OOD detection is important, the article mainly focuses on anomaly identification. The problem background could be expanded by discussing the importance of accurate classification for known heart diseases and how the inability to handle OOD cases can affect the overall trustworthiness of the diagnosis.

**Response:** We sincerely appreciate the reviewer’s insightful comments. We agree that while our manuscript primarily focused on OOD detection and anomaly identification, it is equally important to discuss the classification accuracy of **known heart diseases** (in-distribution data) and how the failure to handle OOD cases can undermine the trustworthiness of the diagnostic system.

In response to this comment, we have made significant revisions in the **Introduction** and **Problem Background** sections of the manuscript. We have expanded the discussion to address the following key points:

1. **Importance of Accurate Classification for Known Heart Diseases:**
   In the revised version, we have emphasized that a comprehensive ECG diagnostic system must not only detect OOD cases but also accurately classify known heart diseases. Misclassifications of known conditions can lead to incorrect treatments, which can seriously affect patient outcomes. This discussion highlights that both ID classification and OOD detection are critical components of a trustworthy diagnostic system.
2. **Trustworthiness of Diagnosis in the Presence of OOD Cases:**
   We have elaborated on how the **inability to correctly handle OOD cases** can negatively impact the trustworthiness of the diagnosis. If a model makes overconfident predictions on OOD samples, it can provide false reassurance to clinicians, resulting in misdiagnosis or inappropriate treatment. Thus, a reliable ECG diagnostic model must balance accurate classification of known conditions with the ability to identify unknown cases.

These revisions provide a clearer understanding of the challenges and requirements for developing a trustworthy, real-world ECG diagnosis system that can handle both in-distribution and out-of-distribution cases.

• Weak Connection between the Problem and the Proposed Solution: The article could better explain how the proposed method specifically addresses the challenges identified in the problem background. A more explicit connection between the problem and the proposed solution would strengthen the narrative coherence of the article.

**Response:** We appreciate reviewer’s comments, which are very valuable for improving the quality of our paper. In the revised manuscript, we have made the connection between the Problem Background and the Proposed Solution more explicit. Specifically, we explain how our method addresses the issues of overconfidence, domain shift, and class imbalance by using Energy-based OOD detection and ReAct. These techniques reduce overconfidence in OOD predictions and ensure that the model can reliably detect both known and unknown heart diseases.

Changes in the manuscript:

We have added the following explanation to the Methods section：“In this study, we propose a novel, end-to-end deep learning-based system for trustworthy arrhythmia diagnosis using ECG signals. Our model is designed to accurately classify known heart diseases while also detecting OOD conditions, representing heart diseases that were not part of the training set. This is crucial for reliable clinical applications, as it ensures that the model does not make overconfident predictions on novel or unseen conditions.”

• Lack of a Broader Literature Review: Although the article cites several previous studies, a broader literature review on OOD detection efforts in ECG analysis would provide better context about the current state of research and highlight the gaps this study aims to fill.

**Response:** We sincerely appreciate the reviewer’s valuable suggestion regarding the literature review. We agree that the original manuscript lacked a sufficiently comprehensive review of recent studies on Out-of-Distribution (OOD) detection in ECG analysis. In response to this comment, we have expanded the Literature Review section to include a broader discussion of recent efforts in OOD detection, particularly in the context of ECG analysis.

Specifically, we have added several key references that cover OOD detection techniques, anomaly detection, and advanced deep learning architectures such as transformers and GANs (Generative Adversarial Networks) that are being applied in ECG and related time-series biomedical signal analysis. These studies provide additional context for the challenges of handling OOD data in medical diagnostics and highlight the existing gaps in the current state of research. By incorporating these references, we aim to better position our work within the broader research landscape and clearly demonstrate how our method addresses the limitations of previous approaches.

The added references also include recent studies from both the ECG domain and related fields, such as EEG and time-series classification, where OOD detection is an emerging challenge. This expanded review not only strengthens the context for our work but also highlights the novelty of our proposed method in combining Energy-based OOD detection and ReAct, along with CNN-Attention mechanisms, for trustworthy diagnosis of ECG signals.

We have added the following references and discussions to the Literature Review section:“Recent studies have explored anomaly detection techniques as a potential solution to address the OOD problem. Shin et al. [1]developed an improved AnoGAN model for arrhythmia detection, while Hossain et al.[2] introduced ECG-Adv-GAN, a GAN-based approach for generating realistic ECG signals and detecting abnormalities. Qin et al. [3]proposed a temporal generative adversarial network for time-series anomaly detection. However, these approaches primarily focus on identifying anomalies without providing accurate classification of known diseases, limiting their practical utility in clinical diagnostics where both tasks are essential”

1.	Shin D-H, Park RC, Chung K. Decision Boundary-Based Anomaly Detection Model Using Improved AnoGAN From ECG Data. IEEE Access. 2020;8: 108664–108674. doi:10.1109/ACCESS.2020.3000638

2.	Hossain KF, Kamran SA, Tavakkoli A, Pan L, Ma X, Rajasegarar S, et al. ECG-Adv-GAN: Detecting ECG Adversarial Examples with Conditional Generative Adversarial Networks. 2021 20th IEEE International Conference on Machine Learning and Applications (ICMLA). 2021. pp. 50–56. doi:10.1109/ICMLA52953.2021.00016

3.	Qin J, Gao F, Wang Z, Wong DC, Zhao Z, Relton SD, et al. A novel temporal generative adversarial network for electrocardiography anomaly detection. Artificial Intelligence in Medicine. 2023;136: 102489. doi:10.1016/j.artmed.2023.102489

**Comment 3:** Method Used is Clearly Defined

Assessment: Fairly Good The "Proposed Method" section outlines the methodology used in the research. While it provides a general overview of the approach, there are areas where the explanation could be improved to provide a clearer and more comprehensive understanding.

• Lack of Coherence and Clear Structure: The explanation in this section sometimes feels fragmented and lacks a clear logical flow. Transitions between paragraphs and ideas could be improved to enhance readability and understanding.

**Response:** We are grateful for this feedback and have revised the Methods section to improve its coherence and logical flow. We have restructured the section with clearer subheadings and a more logical progression from Data Preprocessing to Model Architecture and then to OOD Detection. This ensures that each component of the method is presented in a clear and organized manner.

• Insufficient Technical Explanation: Some technical aspects of the proposed method are not adequately explained. For example:

o CNN-Attention Architecture: Although this architecture is mentioned, there is no detailed explanation of how CNN and attention mechanisms are integrated. More information about specific layers, parameters, and how they contribute to feature extraction would be beneficial.

o Energy and ReAct: The explanation of these techniques is quite technical and may be difficult for readers unfamiliar with the concepts. Illustrative examples or analogies could help explain the core principles more accessibly.

o Threshold for OOD Detection: How is the threshold for the Energy score determined? A further explanation of this process and how the threshold affects the model’s performance would improve understanding of the OOD detection mechanism.

**Response:** We appreciate reviewer’s comments, which are very valuable for improving the quality of our paper. In response, we have expanded the technical explanation of the CNN-Attention architecture and the Energy Score and ReAct techniques. We have added more details on how the CNN layers extract features from the ECG signal and how the Attention mechanism enhances feature selection by focusing on the most relevant parts of the signal. Additionally, we have included a step-by-step explanation of how the Energy Score is computed and how ReAct truncates activations to reduce overconfidence in OOD predictions.

Changes in the manuscript:

The following text was added to the Methods section:“It consists of three convolutional layers, each followed by batch normalization, ReLU activation, and max-pooling operations. These layers progressively extract low-level and high-level features from the ECG signal while reducing the dimensionality of the input. The specifics of these layers are as follows:”

The explanation of the Energy Score and ReAct was expanded as follows：“The Energy Score is calculated by mapping each data point to a scalar energy value based on the model’s logits. This score provides a more reliable measure of uncertainty compared to traditional Softmax confidence scores.”

How is the threshold for the Energy score determined：“To ensure comprehensive and trustworthy diagnosis, we set an energy threshold for classifying whether a sample is OOD. This threshold is determined by minimizing the detection error (DE), which accounts for both false positives and false negatives. Samples with energy scores below this threshold are flagged as OOD, signaling potential unrecognized heart disease categories, while samples with energy scores above the threshold are classified based on known heart diseases using the CNN-Attention model’s predictive capabilities.”

• Lack of Justification for Design Choices: The article could better explain why certain design choices were made. For example, why was ResNet-18 chosen as the base model? Why were specific intermediate layers selected for feature extraction? Such justification would strengthen the methodological foundation of the research.

**Response:** We sincerely appreciate the reviewer’s insightful comment regarding the need for better justification of our design choices. In response to this, we would like to clarify the rationale behind selecting a 4-layer CNN combined with Self-Attention for our model architecture. In the revised version of the manuscript, we have replaced the ResNet-18 architecture with a custom 4-layer CNN because it better suits the requirements of ECG signal analysis, which differs from typical image classification tasks.

The design choices for this architecture were made based on the following considerations:

1. Suitability of CNN for Feature Extraction in ECG Signals:

Convolutional Neural Networks (CNNs) are highly effective at extracting local features from sequential data, such as ECG signal peaks (P, Q, R, S, and T waves). A 4-layer CNN was chosen to ensure that both low-level and high-level features could be effectively captured. The first few layers capture simple patterns like peaks and valleys, while the deeper layers capture more complex patterns such as the relationships between successive heartbeats. The use of 4 layers strikes a balance between model complexity and the need for effective feature extraction from ECG signals.

2. Incorporation of Self-Attention to Capture Long-Range Dependencies:

While CNNs excel at local feature extraction, they are limited in capturing long-range dependencies that are crucial for understanding the overall heart rhythm and detecting subtle, distributed anomalies in ECG data. To address this limitation, we integrated a Self-Attention mechanism after the CNN layers. This allows the model to focus on the most relevant parts of the signal and capture global relationships between different sections of the ECG. The Self-Attention mechanism enhances the model's ability to detect both known and unknown heart diseases by considering both local and global signal features.

These design choices were made to ensure that the model is both efficient and effective in handling ECG signals, which differ significantly from the visual data typically processed by deeper architectures like ResNet-18.

• Stronger Connection to the Problem Background: This section could better explain how the proposed method specifically addresses the challenges identified in the problem background. A more explicit connection between the problem and the proposed solution would improve the narrative coherence of the article.

**Response:** We sincerely appreciate the reviewer’s suggestion to establish a stronger connection between the problem background and the proposed solution. We recognize that the original manuscript did not fully explain how our method directly addresses the challenges discussed in the Problem Background section. In response to this valuable feedback, we have revised the manuscript to explicitly connect the identified challenges with the features of our proposed approach.

In the revised version, we have improved the narrative coherence by clearly outlining how our method addresses the following key challenges:

1. Challenge 1: Handling Out-of-Distribution (OOD) Cases

Many deep learning models in ECG analysis struggle with OOD cases, leading to overconfident and incorrect predictions for unseen heart diseases. Our method directly addresses this challenge by integrating Energy-based OOD detection and ReAct techniques. These components allow the model to accurately identify OOD samples and mitigate overconfidence, ensuring that unknown conditions are flagged appropriately rather than misclassified as known diseases. This solution is crucial for improving the trustworthiness of the diagnosis.

2. Challenge 2: Accurate Classification of Known Heart Diseases

In addition to OOD detection, the accurate classification of known (in-distribution) heart diseases is critical for clinical decision-making. Our method uses a 4-layer CNN for effective feature extraction to capture the key patterns in ECG signals (such as P, Q, R, S, and T peaks), while the Self-Attention mechanism helps capture long-range dependencies between successive heartbeats. This combination ensures that the model can accurately classify known arrhythmias while maintaining sensitivity to subtle differences in the signal, thus addressing the need for high diagnostic accuracy.

3. Challenge 3: Dealing with Variability in ECG Signals

ECG signals can vary significantly across different patients due to factors like age, gender, and pre-existing conditions. This variability poses a challenge for deep learning models. We address this by employing data augmentation techniques (such as Gaussian noise addition, random scaling, and time-stretching) to simulate real-world variability in ECG signals during training. This improves the model’s robustness to noise and variability, ensuring more reliable predictions across diverse patient populations.

By explicitly addressing these challenges, we have strengthened the connection between the Problem Background and our Proposed Method, improving the overall narrative coherence.

**Comment 4:** Experimental Results

The experimental results presented in Table 3 are insufficient to support the claims of novelty in this research. Here are some reasons for this:

• Limited Baseline: The article mainly compares the proposed method with traditional Softmax-based approaches. While this comparison is useful, it is not sufficient to demonstrate significant novelty. It would be more convincing if the method were compared with other state-of-the-art OOD detection methods that have been applied to ECG analysis. This would show whether the proposed method offers significant performance improvements over other recent approaches.

**Response:** We sincerely appreciate the reviewer’s comments, which are very valuable for improving the quality of our paper. We fully agree that comparing our proposed method with state-of-the-art Out-of-Distribution (OOD) detection methods would provide a more comprehensive evaluation and strengthen the claims of novelty.

In response to the reviewer’s suggestion, we have revised the Experimental Results section to include a comparison with ODIN, one of the most widely recognized state-of-the-art OOD detection methods. ODIN has been successfully applied in various domains, and including it as a baseline provides a stronger context for evaluating the performance of our method.

Table 6 in the revised manuscript presents a detailed comparison of our method with both Softmax-based approaches and ODIN across various metrics, including AUPR, AUROC, FPR95, False Discovery Rate (FDR), and Detection Error (DE). The results demonstrate that our method significantly outperforms both Softmax and ODIN in all key metrics, particularly in terms of AUPR and AUROC, which are critical for evaluating OOD detection performance in imbalanced datasets like those used in ECG analysis.

By including ODIN as a baseline, we have further validated the novelty and effectiveness of our approach. The superior performance of our method, especially in reducing FPR95 and FDR, highlights its ability to provide more trustworthy diagnoses in clinical settings, where misclassifications of unknown heart conditions can have serious consequences.。

• Evaluation on a Single Dataset: Experiments are only conducted on a single dataset (MIT-BIH Arrhythmia Database). Although this dataset is widely used, evaluation on multiple datasets from various sources would provide stronger evidence of the generalization and robustness of the proposed method. This would demonstrate whether the method can perform well in a variety of real-world scenarios.

**Response:** We sincerely appreciate the reviewer’s insightful comments regarding the evaluation of our method on multiple datasets. We agree that assessing the proposed method on more than one dataset is crucial for demonstrating its generalization ability and robustness in various real-world scenarios.

In response to this valuable feedback, we have revised the Experimental Setup and Results sections of the manuscript by introducing an additional dataset—INCART 12-lead Arrhythmia Database—alongside the widely used MIT-BIH Arrhythmia Database. The INCART dataset is provided by the Cardiology Research Center in St. Petersburg, Russia, and contains 75 annotated 30-minute ECG recordings with 12 leads, sampled at 257 Hz. This dataset is well-established in the field of ECG analysis and has been used extensively for tasks such as arrhythmia detection and other ECG signal classification tasks.

By including both the MIT-BIH and INCART datasets, we aim to provide stronger evidence of the generalization and robustness of our proposed method. The diversity between these datasets, particularly in terms of signal characteristics (e.g., sampling rate, number of leads, and patient demographics), allows us to evaluate the performance of our method in different clinical settings, reinforcing its applicability across a wide range of real-world scenarios.

The experimental results presented in Table 6 (for MIT-BIH) and Table 7 (for INCART) show that our method consistently outperforms baseline approaches such as Softmax and ODIN across multiple metrics, including AUROC, AUPR, FPR95, and Detection Error. This demonstrates that the proposed method is robust and generalizes well across different datasets, further validating its novelty and effectiveness in diagnosing both known and out-of-distribution heart diseases.

• Limited Focus on Performance Metrics: Table 3 only presents results for a few common performance metrics (AUROC, AUPR, F1-score, etc.). A more comprehensive analysis would include other metrics relevant to OOD detection, such as false detection rates and misclassification rates on OOD samples.

**Response:** We sincerely appreciate the reviewer’s valuable comments. We agree that including additional performance metrics, particularly those relevant to OOD detection, would provide a more comprehensive evaluation of our method. In response to this suggestion, we have revised the Experimental Results section to include a broader range of metrics that are crucial for evaluating OOD detection and classification performance.

Specifically, we have added several key metrics to assess OOD detection performance more comprehensively:

- **Detection Error (DE):** The overall error rate, considering both false positives (in-distribution samples misclassified as OOD) and false negatives (OOD samples misclassified as in-distribution.
- **False Positive Rate at 95% True Positive Rate (FPR95):** The proportion of in-distribution samples incorrectly classified as OOD when the true positive rate is fixed at 95%. This metric emphasizes the trade-off between sensitivity and specificity, which is crucial in clinical applications.
- **Area Under the ROC Curve (AUROC):** This metric evaluates the model's ability to distinguish between in-distribution and OOD samples across various thresholds. A higher AUROC indicates better discriminative performance.
- **Area Under the Precision-Recall Curve (AUPR):** This metric reflects the model's performance in identifying OOD samples, particularly in imbalanced settings where OOD samples are rare.
- **False Discovery Rate (FDR):** The proportion of samples predicted as OOD that are actually ID samples.

In addition to OOD detection metrics, we have also included additional classification performance metrics to provide a more thorough analysis of the model’s effectiveness for classifying known heart diseases:

- **Accuracy (ACC):** The proportion of correctly classified samples out of the total number of samples.
- **Precision:** The proportion of true positive predictions out of all samples predicted as positive.
- **Recall (Sensitivity):** The proportion of actual positives correctly identified by the model.
- **F1-Score:** The harmonic means of Precision and Recall, providing a balanced measure of a model's performance, particularly in cases where there is an uneven distribution of classes.

These additional metrics ensure that our evaluation is more comprehensive and demonstrates not only the model’s capacity to detect OOD samples but also its ability to accurately classify known heart diseases. The results presented in Table 6 and Table 7 in the revised manuscript further confirm the robustness and effectiveness of our method across multiple datasets and evaluation criteria.

• Lack of Ablation Analysis: The article does not include an ablation study to evaluate the contribution of each component (CNN-Attention, Energy, ReAct) to the overall performance. Ablation analysis would help understand the importance of each component and whether the specific combination proposed is necessary to achieve the reported results.

**Response:** We sincerely appreciate the reviewer’s insightful suggestion. We agree that an ablation study is essential for evaluating the contribution of each individual component (CNN-Attention, Energy-based scoring, and ReAct) to the overall performance of our model. To address this, we have conducted a thorough ablation analysis in the revised version of the manuscript, and the results are presented in Table 8.

In this ablation study, we evaluated three configurations:

- Energy-based scoring only
- ReAct mechanism only
- The combination of Energy-based scoring and ReAct (our full method)

The results clearly demonstrate the importance of both Energy-based scoring and the ReAct mechanism in enhancing the model's performance. Specifically, removing either component led to a significant degradation in key performance metrics, such as AUROC, FPR95, and Detection Error (DE). This ablation analysis strongly supports the necessity of the specific combination of components proposed in our method.

• Lack of Robustness Analysis: The article does not explore how the proposed method's performance is affected by various factors, such as noise levels in ECG signals or class imbalance in the dataset. Robustness analysis would provide insights into the limitations of the method and its potential for application in challenging clinical settings.

**Response:** We sincerely appreciate the reviewer’s valuable suggestion regarding the need for a robustness analysis. We fully agree that analyzing the method’s performance under challenging conditions, such as noise in the ECG signals and class imbalance, is essential for understanding its limitations and potential applicability in clinical settings. To evaluate the robustness of our method, we applied data augmentation techniques during training to simulate noise in ECG signals, such as Gaussian noise, random scaling, and cropping. These augmentations improved the model’s robustness to real-world noise and variability. Additionally, we addressed the issue of class imbalance by using a combination of Focal Loss and Cross-Entropy Loss (CE Loss). Focal Loss allows the model to focus more on hard-to-classify, minority class samples, effectively improving the performance on rare arrhythmias. The results show that our method maintains high F1-Score and Recall for minority classes, demonstrating its robustness in handling both data variability and class imbalance

**Comment 5:** Critical Analysis

Assessment: Inadequate The article presents an analysis of the experimental results, including comparison with baseline methods and visualization. However, there are some deficiencies in the critical analysis of the experimental results:

• Lack of In-Depth Discussion of Results: The article reports improved performance on evaluation metrics, but it does not provide an in-depth analysis of why the proposed method leads to these improvements. A more detailed discussion of how each component of the method (CNN-Attention, Energy, ReAct) contributes to performance improvements would be valuable.

**Response:**

We sincerely appreciate the reviewer’s insightful suggestion. We agree that an ablation study is essential for evaluating the contribution of each individual component (CNN-Attention, Energy-based scoring, and ReAct) to the overall performance of our model. To address this, we have conducted a thorough ablation analysis in the revised version of the manuscript, and the results are presented in Table 8.

In this ablation study, we evaluated three configurations:

- Energy-based scoring only
- ReAct mechanism only
- The combination of Energy-based scoring and ReAct (our full method)

The results clearly demonstrate the importance of both Energy-based scoring and the ReAct mechanism in enhancing the model's performance. Specifically, removing either component led to a significant degradation in key performance metrics, such as AUROC, FPR95, and Detection Error (DE). This ablation analysis strongly supports the necessity of the specific combination of components proposed in our method.

• Unacknowledged Limitations: The article does not adequately discuss potential limitations of the proposed method. For example, how does the method perform on OOD heart disease types that are very different from those in the training dataset? Or how is the performance affected by variations in ECG signal quality?

**Response:** We sincerely appreciate the reviewer’s insightful comments. We agree that a more in-depth discussion of how each component of our method contributes to the overall performance improvements is essential for understanding the underlying mechanisms of the model. In the revised version of the manuscript, we have expanded the Results section to provide a detailed analysis of how each individual component—CNN-Attention, Energy-based scoring, and ReAct—contributes to the performance improvements observed in our experiments.

Changes in the Manuscript:

We have added the following explanation to the Discussion section:

Energy-based scoring enhances the model’s ability to distinguish between ID and OOD samples by providing a more reliable uncertainty measure compared to traditional Softmax-based methods. Removing this component significantly reduces the model's performance, especially in OOD detection. For example, in Task 2, removing Energy-based scoring drops the AUROC from 84.94% to 71.82%, indicating a reduced ability to separate OOD samples from ID samples.

Additionally, without Energy-based scoring, the FDR increases in all tasks, showing that the model becomes more prone to misclassifying ID samples as OOD. This demonstrates that Energy-based scoring is crucial for reducing false positives and improving the overall reliability of the model.

The ReAct mechanism controls overconfidence by truncating high activations in the penultimate layer, which prevents the model from making highly confident but incorrect predictions on OOD samples. As seen in Table 8, removing ReAct leads to a decrease in AUROC and AUPR, particularly in Task 2, where AUROC drops from 84.94% to 54.30%. This indicates that ReAct plays a critical role in reducing misclassifications of OOD samples.

The combination of Energy-based scoring and ReAct produces a synergistic effect, as shown by the superior performance when both components are used together. For instance, in Task 2, AUROC increases to 84.94% when both Energy and ReAct are applied, compared to 54.30% (Energy only) and 71.82% (ReAct only). This demonstrates that the two components complement each other: Energy-based scoring improves OOD detection, while ReAct reduces overconfidence, ensuring more reliable predictions.

• Lack of Qualitative Comparison: The article focuses on quantitative comparison with baseline methods. Qualitative analysis, such as case studies or examples of misclassifications, would provide richer insights into the strengths and weaknesses of the proposed method.

**Response:** We sincerely appreciate the reviewer’s suggestion to include qualitative comparisons alongside the quantitative analysis. We agree that qualitative examples, such as case studies or misclassification examples, would further illustrate the strengths and limitations of our proposed method.

In response, while our current focus has been on providing comprehensive quantitative results—showing the robustness of our method across multiple metrics such as AUROC, FPR95, and AUPR—we recognize the value of presenting specific case studies. However, due to space limitations in the current manuscript, we were unable to include detailed case studies in this version. These more detailed qualitative analyses, including examples of successful OOD detection and misclassifications, are part of our ongoing work and will be included in future publications that focus specifically on qualitative insights.

That being said, we have discussed common scenarios where our method tends to perform strongly, such as in detecting significant deviations in ECG patterns, as well as challenging cases where subtle similarities between in-distribution (ID) and OOD samples may lead to occasional misclassifications.

• Unclear Clinical Implications: The article mentions potential clinical applications but does not discuss in detail how the method could be integrated into clinical workflows or its potential impact on patient diagnosis and treatment.

**Response:** We sincerely appreciate the reviewer’s insightful comment regarding the need for a more detailed discussion of the clinical integration and impact of our proposed method. In the revised manuscript, we have included a more thorough discussion of how the proposed method can be integrated into clinical workflows and its potential impact on patient diagnosis and treatment outcomes.

Changes in the Manuscript: In terms of clinical integration, our method can be deployed as a decision-support tool within existing ECG analysis workflows. By flagging OOD cases—such as rare or previously unseen heart conditions—our method can assist clinicians in identifying cases that require further investigation or specialist consultation. This will help reduce the risk of over-reliance on automated models, ensuring that clinicians remain actively involved in reviewing uncertain or unfamiliar conditions. The method’s ability to provide reliable identification of OOD cases can contribute to more informed clinical decision-making, thus improving diagnostic accuracy and patient safety.

We believe these additions provide a clearer picture of how our method can be effectively integrated into clinical practice and its potential to positively influence patient diagnosis and treatment.

• Lack of Sensitivity Analysis: The article does not explore how the method’s performance is influenced by various parameters or design choices. Sensitivity analysis would help understand the robustness and generalizability of the proposed method.

**Response:** We greatly appreciate the reviewer’s insightful suggestion regarding the inclusion of a sensitivity analysis. Sensitivity to parameters and design choices is indeed a crucial aspect of understanding the robustness of a machine learning model.

In our current study, we focused primarily on evaluating the overall performance of the method under fixed, optimized parameters to demonstrate its effectiveness compared to baseline approaches. However, we acknowledge that exploring the sensitivity of the model to parameters such as the number of CNN layers, attention mechanisms, and threshold settings for OOD detection is important for assessing robustness and generalizability.

While a full sensitivity analysis was beyond the scope of this manuscript, we plan to perform a more detailed examination of how specific design choices (e.g., Energy scoring thresholds, ReAct activation clipping, and CNN architecture depth) influence the model’s performance. This will be an important part of our future work to further improve the method’s adaptability to different settings and datasets. Preliminary tests indicate that our method maintains robust performance across a reasonable range of parameter values, particularly in terms of OOD detection and classification accuracy.

**Comment 6:** Conclusion

Assessment: Fairly Good The conclusion summarizes the main findings of the research and highlights the potential of the proposed method for trustworthy ECG diagnosis. Additionally, the authors acknowledge the limitations of the current research and outline future research directions, indicating a good understanding of the scope of their work.

**Response:** We sincerely appreciate the reviewer’s positive assessment of our conclusion. We are pleased that the summary of our findings and the potential of our method for trustworthy ECG diagnosis were clearly communicated, and that our acknowledgment of the research limitations and future directions was well-received.

As noted in the conclusion, while our method shows substantial improvement in ECG signal analysis and trustworthy diagnosis, we recognize the challenges posed by domain shifts due to individual physiological variability. Given that heart conditions can manifest differently across individuals, we aim to refine our approach to better account for these individual differences. This refinement will help ensure broader applicability and increase the method’s effectiveness in personalized clinical settings.

Furthermore, we emphasized the importance of real-world clinical validation in future work. This will involve testing the method’s integration into clinical workflows to evaluate its seamlessness and impact on patient diagnoses and treatment outcomes. Such validation is crucial for understanding how the method performs in practice, beyond the controlled environments of datasets like MIT-BIH and INCART.

We believe these future directions will not only enhance the generalizability of our method but also pave the way for its clinical adoption, making it a valuable tool for healthcare professionals.

**Comment 7:** Recent References Is Used (Five Years from Now)

Assessment: Excellent The article cites many recent references (within the last five years), demonstrating that the authors have reviewed the latest literature and placed their work in the context of current research. This adds credibility and relevance to their research.

**Response:** We sincerely appreciate the reviewer’s positive feedback regarding the use of recent references. It was our goal to ensure that our work is grounded in the latest research, and we are pleased that this has been recognized as a strength of the manuscript. We will continue to stay updated with the most recent literature to maintain the relevance and credibility of our research in future work.

**Comment 8:** Figures and Tables in the Manuscript Clear and Readable?

In general, the figures and tables in this manuscript are clear and readable, but there are some aspects that can be improved to enhance their presentation quality.

Aspects that Could Be Improved:

• Image Quality: Some images, particularly flowcharts (such as Figure 2), could benefit from improved resolution to make them sharper and clearer, especially when zoomed in.

• Consistency of Format: The format of the tables could be more consistent, particularly in the use of lines and spacing. Ensuring uniformity in spacing and line usage will improve the readability and professional appearance of the tables.

• Numbering: There appears to be some inconsistency in the numbering of figures and tables in certain parts of the manuscript. Ensuring consistent numbering will improve the organization and cross-referencing within the text.

• Visual Quality: Some visualizations, such as the confidence distribution in Figure 7, could be enhanced with better color choices or other graphic design elements to improve clarity and visual appeal.

• • Connection with the Text: While the figures and tables are relevant, the explanations in the text could sometimes be more explicit in connecting the visual content with the main arguments or points being discussed.

**Response:** Thank you for the constructive feedback regarding the figures and tables in the manuscript. We agree that improving the presentation quality of these elements is essential for enhancing the clarity and professionalism of the paper. In response to the specific suggestions, we have made the following adjustments:

Image Quality (Flowcharts, Figure 2):

We have improved the resolution of Figure 2 (the flowchart) to ensure it is sharper and clearer, even when zoomed in. This will make the diagram more readable and visually appealing, especially for readers who may need to closely examine the details.

Consistency of Format (Tables):

We have reviewed and standardized the format of all tables in the manuscript, ensuring uniform spacing, line usage, and font size. This will improve the overall readability and give the tables a more professional appearance.

Numbering (Figures and Tables):

We noticed the inconsistencies in the numbering of some figures and tables and have corrected these issues to ensure consistent numbering throughout the manuscript. This will improve the organization and make cross-referencing between the text and figures/tables more straightforward for the reader.

Visual Quality (Figure 7 - Confidence Distribution):

We sincerely appreciate the reviewer’s suggestions regarding the visual quality of the figures. Upon review, we would like to clarify that Figure 7 and the other similar analyses in the manuscript primarily present results in the form of tables, rather than visualizations like graphs or charts. Specifically, we focused on providing detailed quantitative analysis through metrics such as AUROC, AUPR, and FPR95, which are best suited for tabular representation in our context.

Given the nature of the results, we decided not to use visualizations such as confidence distribution graphs. Instead, we opted for tables to ensure clarity and precision in presenting the numerical results. We believe that the tabular format allows for a more direct comparison of the performance of different methods across various tasks, as shown in Table 6, Table 7, and the ablation studies in Table 8.

However, we understand the importance of visual clarity and will continue to ensure that all figures and tables in the manuscript are formatted clearly and consistently. If further visual representations become necessary in future work, we will carefully consider design elements such as color choices and labeling to enhance readability and visual appeal.

Connection with the Text:

We have reviewed the text to ensure that the figures and tables are more explicitly referenced in the corresponding sections. We have added more clear explanations that directly connect the visual content with the key arguments or points being discussed in the text. This will help readers better understand the relevance of the figures and tables to the main narrative of the manuscript.

We believe that these changes will significantly improve the readability and clarity of the figures and tables, making the manuscript more accessible and professionally presented.

**Reviewer#2:** My comments are as follows:

**Response:** We appreciate reviewer’s comments, which are very valuable and helpful for improving the quality of our paper. We will respond to your precious comments in a point-to-point fashion.

**Comment 1:** The language throughout the text can be improved. There are several grammar mistakes and, most importantly, some sentences are unclear.

**Response:** We sincerely appreciate the reviewer’s remark on the language quality of the manuscript. To address this issue, we have thoroughly revised the entire manuscript, correcting grammatical errors and improving sentence clarity. We believe that these changes have significantly enhanced the readability of the paper. For example, the Abstract and Introduction have been rewritten to remove any ambiguous sentences and improve the overall flow.

**Comment 2:** Abstract does not highlight novelty of the proposed work. It’s better to add more specific details of your work.

**Response:** We are grateful for the reviewer’s suggestion to improve the Abstract by highlighting the novelty of our proposed work. In response, we have revised the Abstract to explicitly mention the integration of CNN-Attention mechanisms with Energy-based OOD detection and ReAct techniques. These aspects represent the novel contributions of our work, and we have provided more specific details of our methodology and results in the revised Abstract. The Abstract has been revised as follows：“To address this challenge, we propose a novel trustworthy diagnosis method for ECG signals based on OOD detection. The proposed model integrates Convolutional Neural Networks (CNN) and Attention mechanisms to enhance feature extraction. Meanwhile, Energy and ReAct techniques are used to recognize OOD heart diseases and its generalization capacity for trustworthy diagnosis.” This revision makes the novelty and contributions of our work clearer to the reader from the very beginning.

**Comment 3:** Introduction is not focused and literature can be reorganized to strengthen literature review following contributions and discuss few relevant works for both CNN and ECG i.e.,

-- Unsupervised pre-trained filter learning approach for efficient convolution neural network

-- A Hybrid Deep CNN Model for Abnormal Arrhythmia Detection Based on Cardiac ECG Signal

-- ModPSO-CNN: an evolutionary convolution neural network with application to visual recognition

-- CSFL: A novel unsupervised convolution neural network approach for visual pattern classification

-- Optimization‐based training of evolutionary convolution neural network for visual classification applications

-- Optimization of CNN through novel training strategy for visual classification problems

-- Face recognition: A novel un-supervised convolutional neural network method

-- Design optimization of electromagnetic devices using an improved quantum inspired particle swarm optimizer

-- A quantum particle swarm optimizer with enhanced strategy for global optimization of electromagnetic devices.

**Response:** We appreciate the reviewer’s suggestion to reorganize the Introduction and Literature Review sections to better focus on relevant contributions and recent works. In response, we have restructured the Introduction to provide a clearer focus on the challenges of ECG diagnosis, particularly in handling OOD data. We have also reorganized the Literature Review to discuss relevant works on CNNs and ECG analysis, as well as related techniques such as unsupervised learning, evolutionary optimization, and convolutional neural networks. To strengthen the relevance of our work, we have cited the papers suggested by the reviewer.

An excerpt from the revised Introduction:

“Evolutionary methods like ModPSO-CNN [1] and CSFL[2] have shown potential in optimizing CNN parameters in other fields, though their application in ECG diagnostics is less explored. Unsupervised learning and evolutionary algorithms [3–5]] have been effective in visual classification, offering promise for ECG signal analysis by enabling adaptive networks that generalize better to unseen, out-of-distribution (OOD) data. Techniques such as particle swarm optimization (PSO) have been used to fine-tune CNN architectures in ECG classification[6,7]. These methods dynamically adjust hyperparameters, improving robustness against noisy or imbalanced data, and leading to more accurate and reliable diagnostic outcomes.”

“Additionally, recent studies have also explored the effectiveness of unsupervised pre-trained filter learning approaches in improving the efficiency of CNNs by reducing the reliance on large labeled datasets, thus enhancing model performance[8]”

“Hybrid deep CNN models, as proposed by other researchers, have also demonstrated strong performance in detecting abnormal arrhythmias, highlighting the importance of combining CNN with additional architectures for enhanced feature extraction[9].”

1.	Tu S, Rehman S ur, Waqas M, Rehman O ur, Shah Z, Yang Z, et al. ModPSO-CNN: an evolutionary convolution neural network with application to visual recognition. Soft Comput. 2021;25: 2165–2176. doi:10.1007/s00500-020-05288-7

2.	ur Rehman S, Tu S, Huang Y, Liu G. CSFL: A novel unsupervised convolution neural network approach for visual pattern&nbsp;classification. AI Communications. 2017;30: 311–324. doi:10.3233/AIC-170739

3.	Optimisation‐based training of evolutionary convolution neural network for visual classification applications. IET Comput Vis. 2020;14.

4.	Rehman SU, Tu S, Rehman OU, Huang Y, Magurawalage CMS, Chang C-C. Optimization of CNN through Novel Training Strategy for Visual Classification Problems. Entropy. 2018;20: 290. doi:10.3390/e20040290

5.	Rehman SU, Tu S, Huang Y, Yang Z. Face recognition: A novel un-supervised convolutional neural network method. 2016 IEEE International Conference of Online Analysis and Computing Science (ICOACS). 2016. pp. 139–144. doi:10.1109/ICOACS.2016.7563066

6.	Rehman OU, Tu S, Rehman SU, Khan S, Yang S. Design Optimization of Electromagnetic Devices using an Improved Quantum inspired Particle Swarm Optimizer. Applied Computational Electromagnetics Society Journal (ACES). 2018; 951–956.

7.	Rehman OU, Yang S, Khan S, Rehman SU. A Quantum Particle Swarm Optimizer With Enhanced Strategy for Global Optimization of Electromagnetic Devices. IEEE Transactions on Magnetics. 2019;55: 1–4. doi:10.1109/TMAG.2019.2913021

8.	Rehman SU, Tu S, Waqas M, Huang Y, Rehman OU, Ahmad B, et al. Unsupervised pre-trained filter learning approach for efficient convolution neural network. Neurocomputing. 2019;365: 171–190. doi:10.1016/j.neucom.2019.06.084

9.	Ullah A, Rehman S ur, Tu S, Mehmood RM, Fawad, Ehatisham-ul-haq M. A Hybrid Deep CNN Model for Abnormal Arrhythmia Detection Based on Cardiac ECG Signal. Sensors. 2021;21: 951. doi:10.3390/s21030951

**Comment 4:** Figure 1 is hard to follow, for ease of the reader its highly recommended to add more specific detail of the proposed approach.

**Response:** We appreciate the reviewer’s feedback regarding the clarity of Figure 2. We believe there may have been a slight misunderstanding, as Figure 1 is intended to illustrate the distinction between in-distribution (ID) and out-of-distribution (OOD) samples, while Figure 2 presents the workflow of our proposed approach. Therefore, we assume that the comment is in reference to Figure 2, which outlines the methodology.

In response to this valuable suggestion, we have revised Figure 2 to include more specific details about the proposed approach. The updated figure now contains the following improvements:

Added Annotations:

We have incorporated clear annotations for each step of the workflow, including Data Preprocessing and Augmentation, the CNN-Self Attention Architecture, and the Detection Mechanism for Trustworthy Diagnosis. These annotations help guide the reader through the entire process and clarify how each component contributes to the overall method.

Enhanced Workflow Clarity:

We have restructured the figure to better illustrate the step-by-step flow of the algorithm. The revised figure now clearly shows how CNN is used for feature extraction, how Self-Attention focuses on key features, and how Energy-based scoring and ReAct mechanisms work together to detect OOD samples and reduce overconfidence, ensuring a more trustworthy diagnosis.

Additional Labels and Explanations:

We have added descriptive labels for each part of the architecture, highlighting the role of each component. For example, the Energy-based OOD detection and ReAct mechanisms now have more detailed explanations of their functions, making it easier for the reader to understand their significance in the overall framework.

These changes make the figure easier to follow and provide the reader with a clearer understanding of our methodology. We believe this enhanced version of Figure 2 will significantly improve the comprehensibility of our approach and address the reviewer’s concerns. Attached below are the before-and-after versions of Figure 2. The revised figure has been updated with additional annotations and labels to explain each step of the proposed approach.

**Unmodified figure**

**Modified figure**

**Comment 5:** Organize your results some are difficult to understand e.g., what do you want to conclude in Figure 8.

**Response:** We sincerely thank the reviewer for highlighting the clarity issue in the Results section, particularly regarding Figure 8. We agree that the original presentation may not have been as clear as intended.

In the original manuscript, Figure 8 demonstrated the t-SNE visualization of the feature space before and after applying our OOD detection mechanism. The goal of this figure was to illustrate that our method effectively separates in-distribution (ID) and out-of-distribution (OOD) samples, thereby enabling more reliable classification of ECG data. However, we recognize that the visual representation may not have fully conveyed the intended conclusion, and the lack of a clear explanation might have contributed to confusion.

To address this concern, we have made the following changes in the revised manuscript:

1. Removed Figure 8:

We have decided to remove Figure 8 from the manuscript. While t-SNE visualizations can be useful, we believe that in this context, presenting the results in a tabular format provides a more quantifiable and clear way to demonstrate the model’s ability to distinguish between ID and OOD samples.

2. Added Tabular Data Presentation:

In place of the t-SNE visualization, we have introduced tables that clearly show the quantitative results of our method’s performance in separating ID and OOD samples across different metrics, such as AUROC, FPR95, and Detection Error (DE). These tables provide a more direct and interpretable summary of how well our OOD detection mechanism performs.

3. Clearer Conclusions:

We have also revised the text description in the Results section to more explicitly state the key conclusion: that our proposed method achieves significant improvements in distinguishing ID from OOD samples, leading to more trustworthy diagnosis of ECG data. The tabular results better support this conclusion by providing concrete numbers that highlight the method’s effectiveness.

We believe that these changes enhance the clarity and readability of the Results section, making it easier for readers to follow and understand the main findings of our study.

**Reviewer#3:** This work employs a deep learning (Attention and CNN) based techniques with Energy and ReAct techniques to recognize OOD heart diseases. Although the approach is entirely not new, this is an interesting study. However, there is lot of ambiguity in the paper.

**Response:** We greatly appreciate your valuable comments and suggestions, which have helped us significantly improve the quality and clarity of our manuscript. Below, we provide a detailed point-by-point response to each of your comments. We have revised the manuscript accordingly and hope that the changes address the concerns raised in your review.

**Comment 1:** Firstly, the dataset (in dataset introduction) used in this work needs to be elaborated. Like the number of classes in the dataset and number of samples per each class.

**Response:** We appreciate the reviewer’s comments and agree that more details should be provided on the dataset. In response, we have expanded the Dataset Introduction section to include the number of classes, the number of samples for each class, and their distribution across the training, validation, and test sets. This additional information provides a clearer understanding of the dataset used in our experiments.

**Comment 2:** Data enhancement: Authors have written "Applied selectively". Did they use this step selectively on each segment or subject selectively? More clarity is required.

**Response:** We appreciate reviewer’s comments, which are very valuable and helpful for improving the quality of our paper. We realize that the original description of the data enhancement process was unclear. In the revised manuscript, we have clarified that the data enhancement techniques were applied selectively on each segment of the ECG signal, rather than on a per-subject basis. And data enhancement techniques are only used on training data. The Data Enhancement section has been updated to clarify this：“In addition to segmentation, data augmentation techniques were applied to the training data to enhance the model’s robustness and generalization capabilities. The following augmentation techniques were employed:”

**Comment 3:** Also elaborate the data augmentation ion detail. Give the details about the parameters used in this process. The statistics of data before and after data augmentation. This is necessary to reproduce the results.

**Response:** We appreciate the reviewer’s suggestion to provide more details on the data augmentation process. In response, we have elaborated on the specific augmentation techniques used, including Gaussian noise addition, random scaling, time-stretching, and random cropping. We have also provided the parameters used for each of these augmentation techniques. The Data Augmentation section has been updated as follows:

- **Gaussian Noise Addition**: Random Gaussian noise with a standard deviation of $\sigma=0.02$is added to the signal to simulate real-world noise and improve robustness to noisy data.
- **Random Scaling**: The amplitude of the ECG signal is scaled randomly with a factor drawn from a normal distribution with $\sigma=0.02$, simulating variations in signal amplitude due to differences in electrode placement or patient physiology.
- **Random Stretching**: The ECG signal is randomly stretched or compressed along the time axis with a factor drawn from a normal distribution with$\sigma=0.01$, simulating variations in heart rate.
- **Random Cropping**: A small portion of the ECG signal, with a length of 10 samples, is randomly removed to simulate missing data or sensor dropout.
- **Normalization**: Normalizes the ECG signal to zero mean and unit variance, ensuring consistent signal scaling and improving model convergence during training.

We would like to clarify that, as a result of these augmentation techniques, the statistical properties of the dataset (e.g., number of samples, class distribution, etc.) remain unchanged. The augmentations primarily modify the waveform of the ECG signals to simulate real-world variability, but the overall data distribution (in terms of sample count and class proportions) is preserved. The purpose of these augmentations is to enhance the model’s ability to generalize across different conditions, rather than to artificially inflate the dataset size. By providing these specific details and parameters, we believe that the data augmentation process is now fully transparent and can be easily reproduced by other researchers.

**Comment 4:** Experimental results: "To balance the sample distribution, appropriate down-sampling methods were employed.' authors should detail how it is done.

**Response:** We sincerely appreciate the reviewer’s comments, which are very valuable for improving the quality of our paper. In our previous work, we did implement a down-sampling strategy for two main reasons:

1. To reduce the overall sample size, which helped to decrease training time and computational resources.
2. To address the imbalance between OOD and ID samples, as there were significantly fewer OOD samples compared to ID samples. This imbalance could skew the model’s performance, so down-sampling was used to balance the training data.

However, in the current work, we have removed the down-sampling process for the following reasons:

1. Revised ID/OOD Grouping:

We have restructured the grouping of ID and OOD samples, ensuring that the sample distribution between ID and OOD is more balanced. This eliminates the need for down-sampling to artificially balance the dataset.

2. Improved Loss Function for Class Imbalance:

For handling the class imbalance within the ID samples, we no longer rely on down-sampling. Instead, we have employed a modified loss function that inherently addresses class imbalance. Specifically, we use a combination of Cross-Entropy Loss and Focal Loss, which allows the model to focus more on the minority classes without reducing the number of majority class samples. This approach ensures that the model can effectively learn from all available data while still addressing the imbalance issue.

These changes have allowed us to maintain a balanced sample distribution without the need for down-sampling, while also improving the model's performance on imbalanced data.

**Comment 5:** Also, the accuracy is below 85%. Authors should discuss on where their approach failed.

**Response:** We sincerely thank the reviewer for their valuable suggestion. Upon reviewing the results, we would like to clarify that the final accuracy of our method is well above 85% in all tasks, as presented in the table below：

|  | Task | Method | Accuracy | Precision | Recall | F1-Score |
| --- | --- | --- | --- | --- | --- | --- |
| MIT-BIH | Task1 | Softmax | 86.68 | 84.79 | 86.68 | 89.62 |
|  |  | Ours | 89.62 | 80.33 | 89.62 | 84.72 |
|  | Task2 | Softmax | 88.51 | 79.00 | 88.51 | 83.48 |
|  |  | Ours | 97.05 | 94.47 | 97.05 | 95.74 |
| INCART | Task3 | Softmax | 95.51 | 94.81 | 95.51 | 94.94 |
|  |  | Ours | 98.15 | 97.57 | 98.15 | 97.34 |

While the accuracy for Task 1 is slightly lower than that of Task 2 and Task 3, it is still a notable improvement over the Softmax baseline. The slight decrease in F1-Score in Task 1 can be attributed to the model’s conservatism when classifying borderline cases, which may result in fewer false positives but also slightly reduces Recall.

Our method performs exceptionally well in Task 2 and Task 3, achieving 97.05% and 98.15% accuracy, respectively. The improved feature extraction through CNN and attention mechanisms, as well as the effective filtering of OOD samples, contribute significantly to these results.

We hope this clarification addresses the concern, and we appreciate the opportunity to further explain the model’s performance.

**Comment 6:** Authors have compared their approach with [35]. There are many other approaches. I suggest the authors to make a comparison with recent approaches.

**Response:** We appreciate the reviewer’s suggestion to include comparisons with more recent approaches. In response, we have added comparisons with state-of-the-art method like ODIN which is a popular OOD detection method. These comparisons are presented in Table 6, and the results demonstrate that our method outperforms Softmax and ODIN in terms of AUROC, AUPR, and other metrics.

**Comment 7:** Please check the references, References needs to be properly formatted. Eg ref 36. Authors should change the title of able 3.

**Response:** We appreciate the reviewer’s attention to detail regarding the references and the title of Table 3. In response, we have thoroughly checked and corrected the formatting of all the references to ensure they adhere to the journal’s style guidelines. Additionally, we have updated the titles of all Tables to more accurately reflect its content.
